# Bacteria vs. Bacteriophages: Parallel Evolution of Immune Arsenals

**DOI:** 10.3389/fmicb.2016.01292

**Published:** 2016-08-17

**Authors:** Muhammad A. B. Shabbir, Haihong Hao, Muhammad Z. Shabbir, Qin Wu, Adeel Sattar, Zonghui Yuan

**Affiliations:** ^1^MOA Laboratory for Risk Assessment of Quality and Safety of Livestock and Poultry Products, Huazhong Agricultural UniversityWuhan, China; ^2^Department of Basic Veterinary Medicine, Huazhong Agricultural UniversityWuhan, China; ^3^Quality Operations Laboratory, University of Veterinary and Animal SciencesLahore, Pakistan; ^4^National Reference Laboratory of Veterinary Drug Residues and MAO Key Laboratory for Detection of Veterinary Drug Residues, Huazhong Agricultural UniversityWuhan, China

**Keywords:** bacteriophages, mobile genetic elements, CRISPR system, pre-crRNA, anti-CRISPR system

## Abstract

Bacteriophages are the most common entities on earth and represent a constant challenge to bacterial populations. To fend off bacteriophage infection, bacteria evolved immune systems to avert phage adsorption and block invader DNA entry. They developed restriction–modification systems and mechanisms to abort infection and interfere with virion assembly, as well as newly recognized clustered regularly interspaced short palindromic repeats (CRISPR). In response to bacterial immune systems, bacteriophages synchronously evolved resistance mechanisms, such as the anti-CRISPR systems to counterattack bacterial CRISPR-cas systems, in a continuing evolutionary arms race between virus and host. In turn, it is fundamental to the survival of the bacterial cell to evolve a system to combat bacteriophage immune strategies.

## Introduction

Prokaryotes evolve genetic material under the constant influence of, or exposure to, mobile genetic elements (MGE) such as plasmids and bacteriophages. Bacteriophages are the most abundant biological entities on earth (Bergh et al., 1989; Wommack and Colwell, 2000). Proliferation of bacteriophages occurs in a series of events: attachment or adsorption of a virion to the host cell wall, injection of viral genome (DNA or RNA) through the cell membrane, expression of viral genes, viral genome replication in host cell, and, as a final stage, progeny virions are released from the lysed host (Sturino and Klaenhammer, 2004). Another class of MGEs is plasmids. Plasmid DNA resides in host cytoplasm either free or integrated into the host genome sequence. Plasmids can be transferred from a donor cell to recipient cell by transformation, conjugation, and transduction (Llosa et al., 2002; Thomas and Nielsen, 2005). Integrated MGEs account for up to 30% of some bacterial genomes (Casjens, 2003; Busby et al., 2013).

Bacteria and their associated bacteriophages undergo continuous cycles of evolution to generate resistance to each other in both natural and man-made ecosystems (Stern and Sorek, 2011). The bacterial immune mechanisms/systems developed in response to invaders are enormously diverse, and there are potentially many remaining to be discovered. Currently described innate immune mechanisms involve aversion of phage adsorption or blockage of phage DNA entry. When these mechanisms fail, abortive infection (Abi) comes into action and triggers the suicide of infected bacterial cells, resulting in the prevention of phage replication, which also benefits the bacterial population adjacent to the infected cell.

A clustered, regularly interspaced, short palindromic repeat (CRISPR) locus is the only known adaptive immune system in prokaryotes. A short DNA sequence of the phage is integrated into the CRISPR loci and produces sequence-specific immunity against the invading bacteriophage (Hyman and Abedon, 2010; Labrie et al., 2010). Here, we focus on bacterial innate and adaptive immune systems and phage counter-strategies against the CRISPR-cas system.

## Averting Phage Adsorption/Receptor Mutation

In natural environments, bacteriophages confront a vast diversity of organisms, but generally their host range is limited to a single bacterial species (Hyman and Abedon, 2010). To inject DNA into the target cell, bacteriophages must bind to a specific surface receptor of that cell (Hyman and Abedon, 2010; Labrie et al., 2010). Proteins, polysaccharides, or lipopolysaccharides (LPS) present on the cell surface serve as receptors for bacteriophages. Strategies to avert phage attachment include modification of the structure of the receptor via mutation and concealing the receptor with a physical barrier (Labrie et al., 2010; Dy et al., 2014). For example, *Staphylococcus aureus* produces cell wall associated virulence factor (immunoglobulin G-binding protein A) that binds with the Fc fragment of immunoglobulin G (Foster, 2005). Phage adsorption has been shown to improve when bacteria secrete less protein A (Nordström and Forsgren, 1974). Availability of the receptor can be decreased via variation in the phase during which the expression of the receptor is subjected to heritable, reversible switching that ensures the survival of bacteria population heterogeneity. Two component regulatory system (BvgAS) was identified in *Bordetella* by transposon mutagenesis screening (Weiss et al., 1983). *Bordetella bronchiseptica* shows variation between the Bvg^+^ phase, required for pulmonary colonization, and the Bvg^-^ phase. Bacteria can express various virulence and colonization factors, along with the adhesion molecule pertactin, during the Bvg^+^ phase. Temperate phages showing association with *B. bronchiseptica* clinical isolates have been identified that demonstrate the ability to use pertactin as a receptor (Liu et al., 2002).

In many cases, upon infection, lytic phages hydrolyze host DNA, suggesting that the potential for horizontal DNA exchange between an infecting phage and a prophage is significantly lower than in temperate phages (Hendrix, 2003). However, temperate phages have the ability to enter the host cell as a prophage dormant state and replicate along with the bacterial genome. Upon interaction of these phages with the host, they not only modify the bacterial phenotype, affecting its virulence, but also import new genes into the host (De Paepe et al., 2014).

*Vibrio cholerae* is host to various bacteriophages (vibrio phages) including temperate phages (kappa-type phages) and virulent phages (Mukherjee’s cholera phages). These phages were commonly used for *V. cholerae* O1 phage typing (Basu and Mukerjee, 1968). Later, virulent phages were used for the phage typing of *V. cholerae* O1 (Chattopadhyay et al., 1993) and O139 (Chakrabarti et al., 2000). Filamentous phages, which normally do not kill the host, also played a significant role in the evolution of *V. cholerae* biology. These phages act in horizontal gene transfer (HGT) among *V. cholerae* strains (Faruque and Mekalanos, 2003). For effective colonization of the intestinal tract, *V. cholerae* O1 sero-group strains depend on LPS O1 antigen expression. Clinical samples of *V. cholerae* possess virulent phages, rather than temperate phages, and their relationship relies on the expression of O1 antigen in wild type strains. When this antigen is under the influence of phase variations, it averts phage infection (Seed et al., 2012).

*Haemophilus influenza*, a gram negative pathogen, possesses numerous genes that can produce phase variation via slipped strand mispairing (Hood et al., 1996; Bayliss et al., 2004). Genetic mutations often result in a change of repeat number within, or at, promoter sequences. Within the gene lic2A, DNA polymerase slippage of tetra-nucleotide repeats results in the synthesis of surface lipo-oligosaccharides (LOS), which can be involved in changing the receptor composition of the HP1c1 phage to shift between sensitive and resistant phenotypes (Zaleski et al., 2005).

Expression of receptors present on the cell surface can be modified by competing phages. *Pseudomonas aeruginosa* possesses type IV pilus (TFP) as its mechanism of pathogenesis and biofilm formation, which can be modified by lysogenic conversion. A protein designated Tip, encoded by phage D3112 can bind with ATPase of TFP and avert its localization, leading to the loss of surface piliation and also conferring protection from other phages relying on TFP for infective action (Chung et al., 2014).

Phage receptors may be veiled by a capsule. For example, the K1 capsule of *Escherichia coli* interferes directly with phage T7 and prevents its attachment to LPS receptors (Scholl et al., 2005). Along with hiding receptors, bacteria may produce decoys to circumvent phage attachment. In the presence of an outer membrane vesicle (OMV), the level of phage T4 can be reduced, suggesting that OMV shedding into the environment might act as bait to avert phage attachment. If there is no shedding of OMVs, presence of phage T4 will result in an active infection (Manning and Kuehn, 2011).

Host phase variation can have significant impact on phage adaptation. *E. coli* phage Mu possesses Gin recombinase that plays a role in the inversion of the G segment, leading to the expression of tail fiber genes that act in determining the phage host range. Similar systems have been identified in the P1 phage and in other phages (Sandmeier et al., 1992). In some phages having tail fiber operons, shuﬄons are observed that result in multiple inversions along with production of tail fiber types having diverse host specificities (Sandmeler, 1994).

## Blockage of Invader DNA

After the attachment of the bacteriophage to a specific receptor on the surface of a bacterial cell, it injects its DNA into the host cell where it utilizes host mechanisms for replication (Marks and Sharp, 2000). Superinfection exclusion (SIE) systems of bacteria act in blocking invader DNA entry. These systems consist of membrane-associated proteins encoded by phages and protect the lysogenized host from infection by other closely related phages. *Streptococcus thermophilus* contains TP-J34 phages that demonstrate ability to produce the membrane-localized lipoprotein Ltp_TP-J34_, which interacts with the tape measure protein of other phages (Bebeacua et al., 2013). In *Siphoviridae*, since tape measure protein facilitates DNA passage by channel formation, Ltp_TP-J34_ not only blocks the injection process but also reduces incursion of non-infectious phages. A predicted transmembrane protein, gp15, is produced by *E. coli* phage HK97 that inhibits the DNA entry of HK97 along with closely associated phage HK75 (Cumby et al., 2012). Although many DNA blocking SIE systems have been recognized, there is a need to study their mechanisms of activity. In contrast to receptor blocking strategies, SIE systems provide an advantage to the bacterium in protecting specific cells and adjacent populations against phage superinfection. After DNA injection, the infecting phage facilitates the DNA ejection of non-infectious phages.

## Restriction–Modification Systems

If a bacteriophage successfully adsorbs and injects its DNA into the bacterium, various innate defense systems are in place to avert phage replication. Restriction modification (R-M) systems have the ability to cleave invader DNA. Characteristically, R-M systems consist of restriction endonuclease (REase) and a cognate, methyltransferase (MTase) (Tock and Dryden, 2005). The MTase normally does not modify invader DNA, but acts in self DNA methylation at particular recognition sites. In contrast, REase can recognize invader DNA and degrade it into harmless fragments. Restriction–modification systems are divided into four types on the basis of their subunit composition, recognition site, and mechanism of action (Roberts et al., 2003). Phages can resist R-M systems by incorporating the modified bases (Samson et al., 2013). However, some bacteria (e.g., McrBC in *E. coli*) have modification-dependent REases that act only on modified DNA (Stewart et al., 2000). The restriction system (McrBC) was first described in *E. coli*, in 1952 (Luria and Human, 1952). At that time most phage research involved the T-even phages, and McrBC is active against T-even phage variants having non-glycosylated hydroxymethylcytosine (hmC) in their DNA (Revel, 1983). The restriction enzymes are methylation-dependent and cleave the DNA after recognition who does not have the strain-specific modification (Noyer-Weidner et al., 1986).

Phages have evolved various anti-restriction strategies against R-M systems, including the absence of endonuclease recognition sites in their genomes as the result of point mutations. The polyvalent *Staphylococcus* phage K has no Sau3A sites in its dsDNA genome (Krüger et al., 1987; Tock and Dryden, 2005). Restriction–modification system antiviral efficacy is directly related to the number of recognition sites present in a viral dsDNA genome (Wilson and Murray, 1991). Some phages have overcome R-M systems by acquiring the cognate methylase gene in their genomes (McGrath et al., 1999).

## Abortive Infection

A bacterium survives viral attack in the mentioned innate defense systems, but this is not the case in Abi systems. Under the effect of Abi systems, predation by a bacteriophage results in death of the bacterium, thus protecting adjacent bacterial populations. Abi systems are often encoded by MGEs such as plasmids and prophages (Samson et al., 2013). These systems have the ability to act on any stage of bacteriophage development thus preventing its proliferation. In phage lambda, the RexAB system protects lysogenized cells from infection by several other coliphages through inducing loss of membrane potential, resulting in a decreased level of ATP (Snyder, 1995). More than 20 Abis, designated AbiA to AbiZ, have been identified in *Lactococcus lactis*, a bacterium that encounters bacteriophage attack during its extensive use in the fermentation process of cheese making (Chopin et al., 2005). At an early stage in the bacteriophage replication cycle, AbiP plays a role in the disruption of both phage DNA replication and temporal transcription (Domingues et al., 2004). Premature lysis of the infected cell is induced by AbiZ, ensuring incomplete viral assembly along with prevention of release of infectious virions (Durmaz and Klaenhammer, 2007). It has been recently shown that Abi systems are mediated by toxin-antitoxin (TA) systems (Fineran et al., 2009). For example, the AbiE system can prevent phage proliferation by inducing bacteriostasis (Dy et al., 2014).

## Interference During Assembly

Double strand DNA phages typically possess holin-lysin systems that disrupt their growth cycle, which results in viral progeny release through host cell lysis. Endolysins generally do not have intrinsic secretory signals, holins accumulate and form lesions in the cytoplasmic membrane thereby regulating the endolysins (phage encoded) to the peptidoglycan and, at a precise time point, triggering host cell lysis (Shi et al., 2012). Gram positive bacteria contain phage-inducible chromosomal islands (PICI) that act as phage parasites displaying the ability to interfere with phage reproduction (Ram et al., 2012). Among the growing identified family of PICIs is the well-studied *S. aureus* pathogenicity island (SaPI) that carries and spreads virulence factors (Novick et al., 2010). Under normal conditions, SaPIs reside in the bacterial chromosome but, upon infection, with exposure of helper phages, become active and excise, replicate, and package themselves. Currently described SaPIs have the ability to affect helper phage particle assembly as well as packaging of DNA; however, in contrast to other phage-resistant mechanisms, SaPI action depends on progression of intracellular phage development to produce a generation of mature phage particles loaded with SaPI DNA instead of phage DNA (Ram et al., 2014). During Abi defense, the cell dies a result of phage infection, phage reproduction is limited, and SaPIs spread to adjacent cells. SaPIs use various unique strategies to interfere with phage reproduction. They can modify phage capsid protein into small capsids containing the SaPI genome rather than the larger helper phage genome (Ruzin et al., 2001; Ram et al., 2012). Phage packaging interference (Ppi) proteins are encoded by SaPIs that are thought to play a role in blocking the phage terminase small subunit necessary for phage DNA recognition as well as for initiation of packaging, allowing the SaPI terminase small subunit to interact with the phage-encoded large subunit to facilitate SaPI DNA cleavage for packaging (Ram et al., 2012). A further interference mechanism involves interruption in late gene activation of phages, which is necessary for phage packaging as well as cell lysis (Ram et al., 2014). *V. cholerae* contain a PICI-like element, which acts in the inhibition of virulent phages (Seed et al., 2013), although the mechanism of this activity is yet to be discovered.

## Crispr: Bacteria Adaptive Immune System

Bacteria CRISPR-cas systems have the ability to target and destroy DNA of MGEs such as plasmids, phages, transposons, and pathogenicity islands. CRISPR loci and their associated cas genes participate in the adaptive defense mechanism to protect bacteria against invaders (Deveau et al., 2010; Marraffini and Sontheimer, 2010). A CRISPR locus consists of short arrays of repetitive sequences that are separated by plasmid- or bacteriophage-derived spacer sequences. The mechanism of the CRISPR system against invaders DNA consists of three steps: *Adaptation* (Garneau et al., 2010; Marraffini and Sontheimer, 2010) involves the acquisition of a spacer after recognition and discrimination of two repeat units present within the CRISPR array. As the spacers are integrated at the leader end of the CRISPR loci, the position of the spacer in the locus provides a clue to its acquisition event (Deveau et al., 2008). Cas1 and cas2 proteins are essential to adaptation, as they are critical to the acquisition of the spacer.

In the *CRISPR expression* step, pre-CRISPR RNA (pre-crRNA) transcription occurs by RNA polymerase within the CRISPR array. After transcription, the pre-crRNA is cleaved by specific endoribonucleases into small CRISPR RNAs (crRNA). Due to its function, crRNA is also called guide RNA (Brouns et al., 2008; Carte et al., 2008).

**FIGURE 1:**
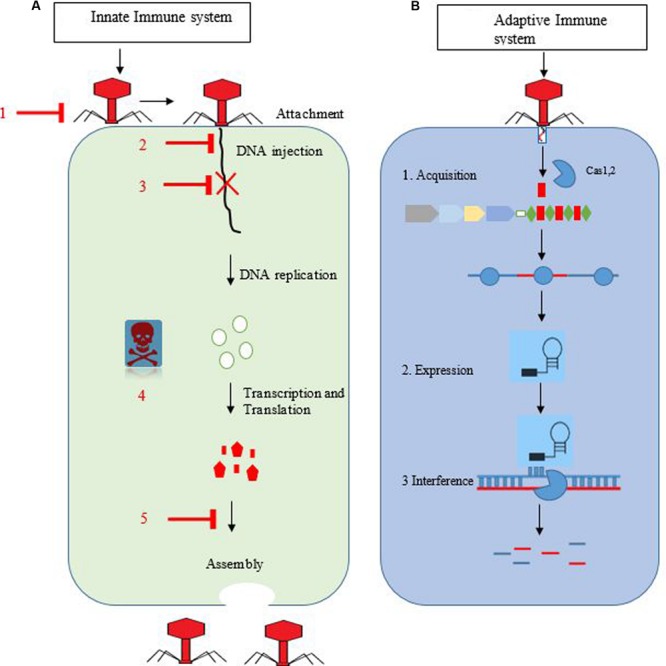
**(A,B)** Overview of the innate and adaptive immune system of bacteria against bacteriophages. Bacterial anti-phage systems with flat arrow heads. 1 = averting phage adsorption, 2 = blockage of invader DNA entry, 3 = restriction–modification system, 4 = abortive infection, and 5 = interference during assembly. The right side represents stages of bacteriophage replication in host cells. **(B)** Represents the CRISPR-cas immune system. In the first stage, spacer is integrated with the help of Cas1,2 proteins into the CRISPR loci. In second stage, biogenesis and maturation of pre-crRNA into mature crRNA occur. In the final stage, foreign DNA is cleaved (blue and red stripes) with the help of a specific enzyme.

In the final step, *interference*, multi-protein complexes having mature crRNA recognize and form specific base pairs with invader DNA or RNA (Brouns et al., 2008), resulting in cleavage of crRNA-invader nucleic acid complex (**Figure 1**) (Garneau et al., 2010).

## Counterattack of Invaders Against the Crispr-Cas System

Phages of *P. aeruginosa* have been found to encode proteins that show ability to inactivate the CRISPR-cas system. The type I-F system is inhibited by five anti-CRISPR protein families, while the type I-E system is inhibited by four anti-CRISPR protein families (Bondy-Denomy et al., 2013; Pawluk et al., 2014). The genes responsible for encoding of these proteins have been observed in Mu-like phages. The anti-CRISPR operon was found to be present in the same position in a variety of *Pseudomonas* related phages. However, the anti-CRISPR gene complement that comprise the locus varies among phages. For example, of 24 related phages displaying the anti-CRISPR operon, 15 encode the anti-CRISPR genes of both CRISPR system types I-E and I-F, one encodes type I-E only, and type I-F only is encoded by other eight (Pawluk et al., 2014). Collectively, nine distinct arrangements were provided by these phages in type I-E and I-F anti-CRISPR genes, revealing that these genes re-assort several times via HGT in a mix-and- match manner. Due to the few homologous sequences, it is difficult to trace the anti-CRISPR protein evolutionary origin. Nevertheless, their frequent occurrence in *Pseudomonas* phages suggests that they provide substantial evolutionary advantage.

## Function of Anti-Crispr Proteins Exhibited By Various Mechanisms

**FIGURE 2:**
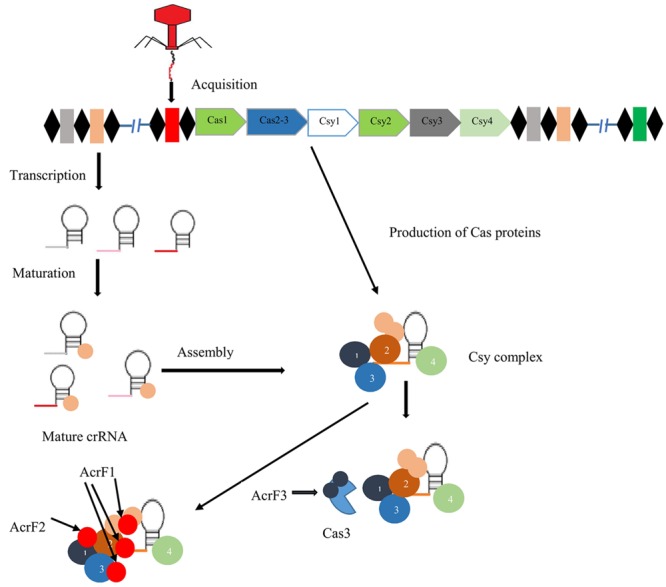
**Mechanism of action of three anti-CRISPR proteins.** Black diamonds indicate direct repeats of CRISPR system type I-F separated by spacer sequences (colored boxes). In the presence of cas1,2 proteins, foreign DNA (red box) is acquired at the CRISPR locus. After that biogenesis of crRNA, mature crRNA forms in response to pre-crRNA transcription. crRNA-Csy complex binding with Csy1, Csy2, and Csy3, results in the formation of surveillance complex. Anti-CRISPR proteins AcrF1 and AcrF2 interact directly with this complex and block the binding of DNA. AcrF3 interacts with Cas3 nuclease protein and prevents its recruitment (Maxwell, 2016).

In response to phage attack, CRISPR system type I-F activity is categorized into three stages (**Figure 2**): In the first stage, invader phage DNA is recognized and incorporated into the CRISPR array as spacer by cas1 and cas2 proteins. In the next stage, transcription of pre-crRNA occurs, which is cleaved into mature crRNA, facilitated by Csy4 endoribonuclease that remains attached to the 3′ end of crRNA (Haurwitz et al., 2010). This crRNA-Csy4 complex interacts with Csy1, Csy2, and Csy3 proteins, resulting in the formation of a Csy complex (Wiedenheft et al., 2011). This complex binds with foreign DNA (complementary to crRNA) by surveying the bacterial cell. Upon binding with foreign DNA, Cas3 helicase–nuclease protein is recruited by this complex resulting in degradation of target DNA (Westra et al., 2012; Huo et al., 2014).

The anti-CRISPR system of type I-F possesses three unique proteins, AcrF1, AcrF2, and AcrF3 (Bondy-Denomy et al., 2015). In the Csy complex, the heterodimer of Csy1-Csy2 bind at the 5′ end of crRNA, while the monomer of Csy4 binds at the 3′ end, and Csy3 (six subunits) binds along the RNA spacer (Haurwitz et al., 2010; van Duijn et al., 2012). AcrF1 and AcrF2 proteins interact with the Csy complex to block DNA binding with this complex (**Figure 2**). AcrF2 is bound to the heterodimer of Csy1–Csy2 with a stoichiometry of one anti-CRISPR molecule per complex. This leads to inactivation of the CRISPR-cas system due to blockage of the 5′ end. It has been demonstrated that AcrF1 binds along the full length of Csy3. Unlike AcrF2, AcrF1 has the ability to bind with the complex even when the complex is already bound to DNA. Hence, both AcrF1 and AcrF2 bind with the Csy complex, but with different mechanisms of action. AcrF3 interacts directly with cas3 endoribonuclease to block of its recruitment to the Csy complex (**Figure 2**). Adaptation of anti-CRISPR mechanisms to diverse protein sequences indicate independent evolutionary pathways.

## Anti-Crispr Genes in MGEs

Survival of MGEs upon invasion into bacteria can be increased if they show the ability to inactivate CRISPR-cas systems. Recently, several anti-CRISPR homologs were found within non-phage-associated genome regions of various *Pseudomonas* strains. These regions include many genes encoding proteins (likely MGEs) that are active in DNA transfer and conjugation (Bondy-Denomy et al., 2013; Pawluk et al., 2014). These anti-CRISPR genes can increase the survival of MGEs upon inter-strain transfer by inactivating the recipient CRISPR-cas systems.

It is also thought that MGEs possessing anti-CRISPR proteins might play a role in enhancing the virulence of bacterial strains. For example, an active pathogenetic area containing anti-CRISPR homologs has been identified in clinical isolates of highly virulent *P. aeruginosa*, and it is believed that this pathogenicity island is transferred via conjugation among *P. aeruginosa* (Battle et al., 2008). The discovery of anti-CRISPR genes in MGEs suggests that they might play a key role in lateral gene transfer by permitting invader DNA to evade CRISPR-cas systems.

## Summary

Bacteriophages exert strong selective pressure that plays a significant role in most ecosystems, not only to control the numbers, but also the composition, of the bacterial population. Likewise, bacteria develop immune strategies to avert phage attack by regulating the number and composition of phages, leading to the establishment of predator-prey dynamic equilibrium. Plasmids and prophages that act as a self-interested elements play are vital to the development of phage resistance strategies. These elements provide effective barriers against phage infection without compromising the biological integrity of the host cell. Phages continually adapt and evolve in response to bacterial immune system evolution. Phages related to *Pseudomonas* have developed anti-CRISPR systems to counterattack the CRISPR-cas system type I-E and I-F present in the bacterial host.

## Future Perspectives

1. There is a need for molecular characterization of anti-phage systems that are yet to be fully understood.
2. The discovery of the *Pseudomonas*-associated phage anti-CRISPR system opens a discussion of whether other bacteria-associated phages have developed similar strategies.

## Author Contributions

MS wrote a manuscript while HH, MS, QW, AS, and ZY reviewed and edited manuscript. All authors read and approved the final manuscript.

## Conflict of Interest Statement

The authors declare that the research was conducted in the absence of any commercial or financial relationships that could be construed as a potential conflict of interest.
